# Cenobamate as add-on therapy for drug resistant epilepsies: effectiveness, drug to drug interactions and neuropsychological impact. What have we learned from real word evidence?

**DOI:** 10.3389/fphar.2023.1239152

**Published:** 2023-12-21

**Authors:** Nicola Pietrafusa, Giovanni Falcicchio, Emilio Russo, Simona Lattanzi, Bianca Goffredo, Raffaele Simeoli, Sara Cairoli, Tiziana Corsetti, Roberta Roberti, Marina De Tommaso, Federico Vigevano, Angela La Neve, Nicola Specchio

**Affiliations:** ^1^ Clinical and Experimental Neurology, Full Member of European Reference Network EpiCARE, Bambino Gesù Children’s Hospital, IRCCS, Rome, Italy; ^2^ Department of Translational Biomedicine and Neurosciences, University of Bari ‘Aldo Moro’, Bari, Italy; ^3^ Science of Health Department, University Magna Grecia of Catanzaro, Catanzaro, Italy; ^4^ Neurological Clinic, Department of Experimental and Clinical Medicine, Marche Polytechnic University, Ancona, Italy; ^5^ Division of Metabolic Diseases and Drug Biology, Bambino Gesù Children’s Hospital, IRCCS, Rome, Italy; ^6^ Hospital Pharmacy Unit, Bambino Gesù Children’s Hospital, IRCCS, Rome, Italy; ^7^ Head of Neurological Sciences and Rehabilitation Medicine Scientific Area, Bambino Gesù Children’s Hospital, IRCCS, Rome, Italy

**Keywords:** cenobamate, epilepsy, focal-onset seizures, drug-resistance, blood levels, neuropsychology

## Abstract

**Background:** Cenobamate (CNB) is an anti-seizure medication (ASM) approved in 2021 in Europe for adjunctive treatment of focal-onset seizures in adults who were not adequately controlled with at least two previous ASMs.

**Methods:** seizure outcome, treatment-emergent adverse events, neuropsychological profile, and blood levels of CNB and concomitant ASM were analyzed in a real world setting in two different Italian epilepsy centers in the context of CNB early access program. All patients performed a general cognitive evaluation, while 32 patients underwent the administration of a battery of neuropsychological tests at baseline and 6 months after CNB treatment. We performed CNB quantification in plasma in 31 patients at different doses in the range of 100–400 mg/day (65 measures).

**Results:** we enrolled 54 patients with a median age of 27.9 years. The mean follow-up was 10.7 months. Most (91%) completed the efficacy analysis. At last follow-up visit, a 69.5% median seizure reduction was registered. Thirty-two patients (59.2%) had a ≥50% reduction of seizures that was ≥75% in 20 (42.0%) cases, whilst 10 (20.2%) patients were seizure-free. The most common adverse events were somnolence (53.1%), dizziness (28.1%) and diplopia (12.5%). The correlation between CNB dose and plasma concentration, revealed a significant linear correlation (*r* = 0.86, *p* < 0.0001), and there was a significant difference in mean plasma concentration/dose administered ratio (C/D ratio) between patients taking or not at least one inducer (0.10 ± 0.04 [(μg/mL)/(mg/day)]; *n* = 47 vs*.* 0.13 ± 0.05 [(μg/mL)/(mg/day)]; *n* = 18, *p* = 0.04). CNB dose was inversely correlated (*r* = −0.31, *p* = 0.02) to the C/D ratio of Carbamazepine blood levels. and positively correlated (*r* = 0.74, *p* < 0.0001) with an increased plasma concentration of the active Clobazam metabolite N-desmethylclobazam. General Anxiety Disorder-7 showed a significant improvement of score from baseline evaluation of 6.82 to follow-up 6 months evaluation of 4.53 (*p* = 0.03).

**Conclusion:** In this real-world study, we registered a clinically meaningful reduction in seizure frequency after CNB administration in most patients along with a good tolerability profile. CNB treatment is correlate to a reduction in symptom severity of anxiety score. Plasma levels measurements confirm that CNB acts both as “victim” and as “perpetrator” of drug-drug interactions.

## Introduction

In the last decades, numerous anti-seizure medications (ASMs) with different mechanisms of action have been marketed representing new therapeutic alternatives for clinicians and for patients with epilepsy (Perucca et al., 2020). Despite the early use of new ASMs, up to 30% of patients will have inadequate seizure control (Kwan and Sander, 2004; Mohanraj and Brodie, 2005) with the chances to reach seizure freedom being lower with each treatment failed (Chen et al., 2018). The severity of epilepsy results in an urgent need for developing new and more effective pharmacologic treatments (Perucca et al., 2020). Cenobamate (CNB) is the latest ASM approved as adjunctive treatment for focal-onset seizures (FOS) in adult patients who have not been adequately controlled despite a history of treatment with at least two ASMs (Roberti et al., 2021). CNB has multiple mechanisms of action: it mainly inhibits the persistent component of the sodium current trough the inactivation of the neuronal voltage-gated sodium channels (Nakamura et al., 2019), and acts as a positive allosteric modulator of the γ-aminobutyric acid (GABA_A_) ion channel binding at a non-benzodiazepine site (Sharma et al., 2020). Adjunctive CNB in adult patients with uncontrolled FOS has been associated with a greater reduction in seizure frequency than placebo in two pivotal multicenter, randomized, double-blind, placebo-controlled trials enrolling 659 patients (Lattanzi et al., 2020; Specchio et al., 2021). Interestingly, CNB treatment has been associated with seizure freedom rates which are considerably higher than those reported for other ASMs (Villani et al., 2022). A phase III, open-label, safety study (Sperling et al., 2021) further documented the safety of CNB when the drug is started at a lower dose (12.5 mg/day) and up-titrated bi-weekly. To date, only few real-world studies evaluated the efficacy and safety of CNB. Recently published series provided evidence about the efficacy and safety of CNB in both pediatric and adult patients, including patients with developmental and epileptic encephalopathies (DEEs) (Elliott et al., 2022; Makridis et al., 2022; Varughese et al., 2022; Beltrán-Corbellini et al., 2023; Friedo et al., 2023; Peña-Ceballos et al., 2023; Villanueva et al., 2023).

While data of safety and effectiveness are available, data on effects of CNB on neuropsychological functions are still lacking, moreover drug to drug interactions and blood levels of CNB are also missing. In the present study, we report the effectiveness, tolerability, neuropsychological outcomes, and occurrence of drug-drug interactions through the analysis of the CNB blood levels in a cohort of drug-resistant adult patients treated with CNB as adjunctive treatment.

## Methods

### Study design and population

We performed a retrospective study of consecutive patients treated with CNB attending two epilepsy centers, i.e., Rare and Complex epilepsy Unit of Bambino Gesù Children Hospital (Rome, Italy) and the Epilepsy Center of Bari University Hospital (Policlinico of Bari, Italy). All patients received CNB through Angelini Pharma’s Early Access Program (EAP) between 2020 and 2022. We included all patients treated with add-on CNB for, aged ≥18 years and diagnosed with drug-resistant focal epilepsy. Patients with severe hepatic impairment or end-stage renal disease (including patients on hemodialysis), history of suicidal attempt, drug abuse or alcoholism, psychogenic or non-epileptic seizures and women with known (or planned) pregnancy were excluded. Written informed consent was obtained from patients or their legal representatives.

Patients underwent clinical evaluation every 3 months according to routine clinical practice or whenever clinically indicated (i.e., occurrence of adverse events [AEs] or need of therapeutic adjustments). Demographic, seizure- and treatment-related data were obtained from medical records and seizure diaries. CNB was started at the dose of 12.5 mg/day and up-titrated according to prescribing information until 200 mg/day. The maximum allowed daily dose was 400 mg/day. Duration of the titration period and CNB maximum achievable dose were at the epileptologists’ discretion. Changes in the titration schedule of CNB and in the doses of concomitant ASMs were allowed if AEs occurred.

### Assessment of efficacy

The 4 weeks before starting CNB were identified as the baseline period. Efficacy outcomes were evaluated at 3, 6, 9 and 12 months after the introduction of CNB. The primary efficacy endpoint was the percentage change in monthly baseline seizure frequency at the last follow-up visit (LFV). The seizure frequency up to the LFV was calculated as the number of seizures recorded during the treatment with CNB (including both the titration and maintenance phase), divided by the number of days from the initiation of CNB to the LFV; the result was multiplied by 28 to obtain a monthly frequency. Both median and mean percentage change in monthly baseline seizure frequency at the LFV were calculated using the following formula: (seizure frequency through LFV − seizure frequency during baseline) × 100/seizure frequency during baseline.

Secondary efficacy endpoints were the percentages of patients who had ≥25%, ≥50%, ≥75%, or 100% reduction in baseline monthly seizure frequency at the LFV and over 3-month intervals (0–3; 3–6; 6–9; and 9–12 months). Seizure freedom was defined as no seizures since the prior visit. Patients with ≥50% reduction in baseline seizure frequency were defined as responders. Reduction in monthly seizure frequency <25% or any increase in baseline seizure frequency were also reported. The proportions of patients who were seizure-free at LFV or had no more than one seizure for six consecutive months were also considered.

### Assessment of tolerability

Tolerability was summarized every 3 months. The number and percentage of individuals reporting AEs were recorded, considering the supposed causal relationship with the study drug. AEs were classified as mild (not interfering with normal everyday activities), moderate (interfering with normal everyday activities), or severe (preventing normal everyday activities). Serious AEs (SAEs) were summarized separately.

Considering the predicted higher incidence of AEs in patients tacking sodium channel blockers (SCBs) or clobazam, data was also analyzed comparing such subgroups (Table 5).

### Neuropsychological evaluation

Neurocognitive outcomes were assessed at baseline and every 6 months through self- or parental-administered instruments: Progressive Matrices 38 IQ, the Trail Making Test (TMT), the Adaptive Behavior Assessment System, 2nd edition (ABAS-II), PHQ-9 (Patient Health Questionnaire), GAD-7 (Generalized Anxiety Disorder), Pediatric Quality of Life Inventory (PEDsQL) and QOLIE-31 (Quality of life in epilepsy-31 inventory).

### Measurement of cenobamate plasma concentration by LC-MS/MS and other ASMs

Plasma concentrations of CNB and concomitant ASMs were performed every 3 months. Plasma laboratory analysis has all been performed in Rome (Bambino Gesù Children Hospital). Cenobamate plasma concentration was measured using an ultra-performance liquid chromatography (UPLC) 1,290 Infinity II system (Agilent Technologies) coupled to a 6,470 Mass Spectrometry system (Agilent Technologies) equipped with an ESI-JET-STREAM source operating in the positive ion (ESI+) mode. CNB powder was of analytical grade and was purchased from Spectra 2000 Srl (Rome, Italy). Detailed information on CNB methods for plasma concentration measurements is available as Supplementary Data.

### Statistical analysis

Continuous data were summarized using descriptive statistics including means, standard deviations, medians, lower and upper quartiles, and ranges. Categorical variables were summarized with frequencies and percentages. Univariate comparisons were made by the chi-square test or the Fisher exact test for categorical variables and the Student’s t test or Mann-Whitney U for continuous variables as appropriate; the analysis of variance was used to compare means across multiple groups.

A stepwise binomial logistic regression analysis was used to assess independent associations: in the final model, age at epilepsy onset, disease duration, numbers of previous ASMs, numbers of current ASMs, baseline seizure frequency and presence of focal to bilateral tonic-clonic seizures were the dependent variables and CNB response (≥50% seizure frequency reduction at LFV) was the independent variable. The model was validated by the Hosmer-Lemeshow test, and the stepwise entry method was used. We limited the number of independent variables to a minimum of 10:1 event per independent variable. Statistical significance was set at *p* < 0.05. Statistical analysis was performed using R version 3.2.3 (R Foundation for Statistical Computing, https://www.r-project.org/).

For measurement of plasma concentration of CNB by LC-MS/MS and other ASMs, all statistical analyses and graphs were performed using Graph-Pad Prism 9.0 (Graph-Pad software Inc., San Diego, CA). Cenobamate plasma concentrations were reported as median with range whereas ASMs plasma levels were expressed as median with interquartile range (IQR). D’Agostino & Pearson test was used to check data distribution normality.

## Results

Fifty-four patients (31 F) with a median age of 27.9 years (IQR = 21.7-33.4) were enrolled. The mean follow-up duration was 10.7 months [range ±standard deviation (SD) 6.0-15.0 ± 2.1]. All patients presented FOS, 18 (33.3%) of them suffering from focal to bilateral tonic-clonic seizures. Patients were previously treated with a median of 9 ASMs (IQR = 7–9). When CNB was started, patients were taking a median of three ASMs (IQR = 1–4). The most common concomitant ASMs at the enrollment were carbamazepine (*n* = 24, 44.4%), clobazam (*n* = 22, 40.7%), and lacosamide (*n* = 17, 31.5%). Sixteen patients (29.6%) had vagal nerve stimulation therapy and 19 out of 54 patients (35.2%) had failed epilepsy surgery. Details of demographic and clinical characteristics of the study cohort are shown in Table 1.

**TABLE 1:** Baseline demographics, clinical features (*N* = 54) and Efficacy data (*N* = 49).

	N (%) or Mean (ranges ±SD) or Median (Q1, Q3)
Patients	54
Sex	
Male	23 (42.6)
Female	31 (57.4)
Age at epilepsy onset (years)	5.8 (2.7, 8.9)
Age at enrolment (years)	27.9 (21.7, 33.4)
Disease duration (years)	22.5 (16.1, 28.4)
Etiology	
Unknown	17 (31.5)
Genetic	5 (9.2)
Genetic/Structural (TSC)	3 (5.5)
Structural	26 (48.1)
Infective	2 (3.7)
Autoimmune	1 (1.8)
Previous SE	7 (12.9)
Follow-up (months)	10.7 (6.0-15.0 ± 2.1)
Dose of CNB at last follow-up (mg/day)	201.8 (25-400 ± 96.9)
Weight (kg)	66.6 (30-121 ± 15.7)
Previous idiosyncratic reactions	5 (9.2)
Previous ASMs	9 (7, 12)
Concomitant ASMs (number)	3 (1, 4)
Other treatments	
VNS	16 (29.6)
Neurosurgery	19 (35.2)
KD	0 (0)
CNB dose (mg/day) mean	201.8 (25-400 ± 96.9)
CNB dose (mg/day) median	200 (100, 300)
Seizure type (n, %)	
Focal	54 (100)
Focal to bilateral	18 (33.3)
Other types of seizures	
Tonic	5 (9.2)
Atonic	3 (5.5)
Atypical absences	2 (3.7)
Spasms	2 (3.7)
Baseline seizure frequency	
Mean	23.5 (5-100 ± 25.7)
Median	11.7 (5.5, 31.9)
Titration period (days) at 100 mg/day	58.4 (30-151 ± 18.9)
Titration period (days) at 200 mg/day	107.7 (70-278 ± 40.3)
Titration period (days) at CNB maximum dose	167.9 (14-434 ± 109.7)
CNB withdraws (n, %)	9 (16.7)
Adverse events	5 (9.2)
Inefficacy	2 (3.7)
Increased seizure frequency	2 (3.7)
Last follow-up seizure frequency	
Mean	10 (0-64 ± 15.5)
Median	3.5 (0, 9.7)
Median duration of maintenance dose (months)	4.0 (2.3, 7.0)
Median percentage reduction in baseline seizure frequency at LFV	69.5 (20.8, 98.2)
≥50% reduction in seizure frequency at LFV	29 (59.2)
100% reduction in seizure frequency at LFV	10 (20.4)
N. of pts seizure-free for 6 consecutive months	
Baseline seizure frequency	6 (13.3)
Mean	23.5 (5-100 ± 25.7)
Median	11.7 (5.5, 31.9)
N. of pts nearly seizure freedom (one seizure in 6 consecutive months)	4 (8.9)

N, number; SD, standard deviation; Q1, first quartile; Q3, third quartile; TSC, tuberous sclerosis complex; SE, status epilepticus; CNB, cenobamate; VNS, vagal nerve stimulation; KD, ketogenic diet; LFV, last follow-up visit.

Titration period up to 100 mg/day lasted for a mean period of 58.4 (range ±SD 30-151 ± 18.9) days; 107.7 (70-278 ± 40.3) days was the mean time to reach 200 mg/day and 167.9 (14-434 ± 109.7) days were needed to achieve the maximum dose of CNB (204 mg, 50-400 ± 90). At LFV, the mean dose of CNB was 201.8 mg/day (25-400 ± 96.9). In 9 patients (16.7%) CNB was withdrawn due to AEs (*n* = 5; 9.2%), inefficacy (*n* = 2; 3.7%) or seizure worsening (*n* = 2; 3.7%).

### Effectiveness

**FIGURE 1:**
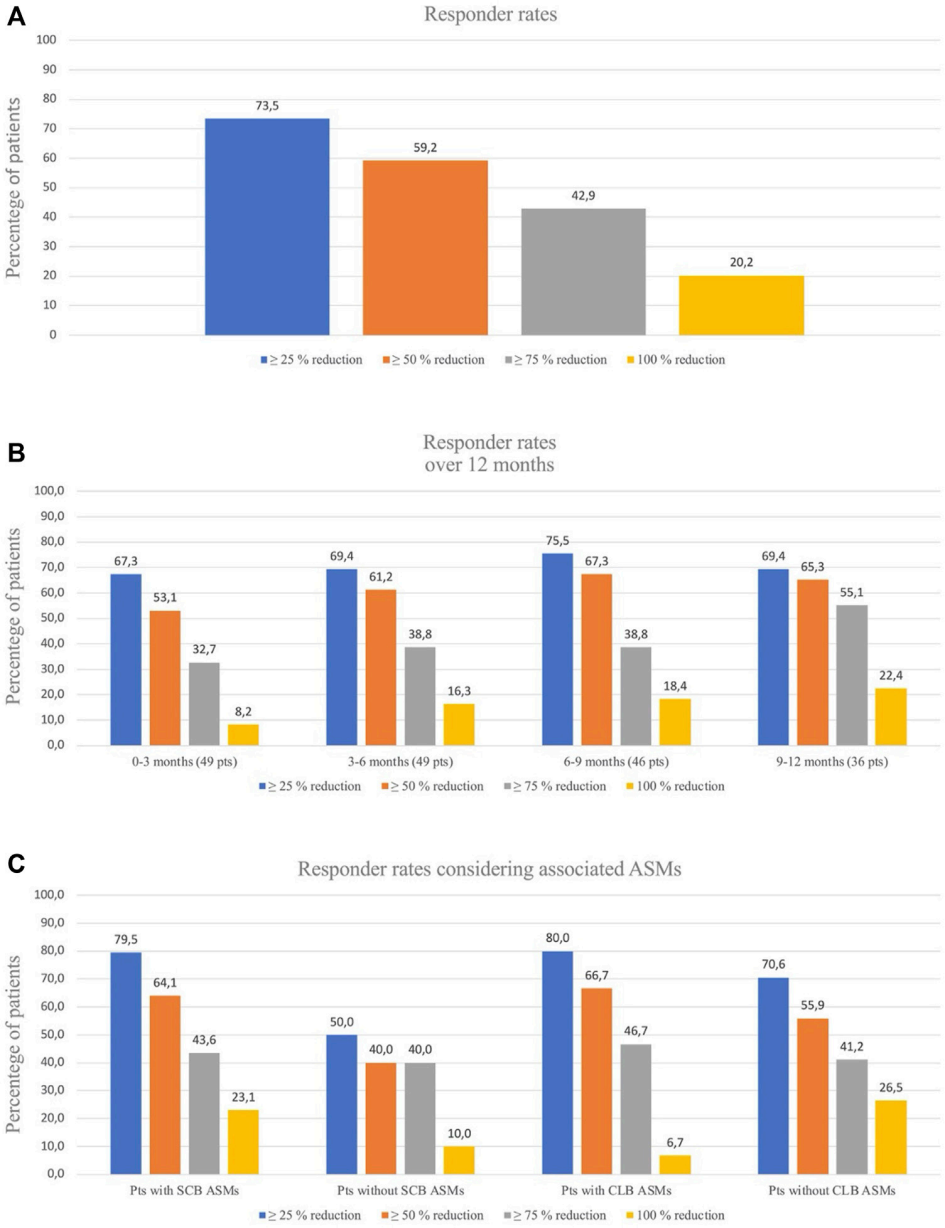
**(A)**. Responder rates for seizures reduction al last follow-up visit. **(B)**. Responder rates over time for seizures frequency. At 0–3 months and 3–6 months, data were missing for 0 patients; at 6–9 months there were missing for 3 patients and at 9–12 months there were missing for 13 patients. **(C)**. Responder rates considering associated ASMs: patients with/without Sodium Channel Blockers (SCB) associated ASMs and patients with/without Clobazam (CLB) associated ASMs.

Data on seizure frequency were available for 49 patients (90.7%). The remaining patients (*n* = 5) dropped-out before 3 months of follow-up and they did not reach the 200 mg dose due to drug withdrawal (2 for seizure worsening and 3 for AEs) and were excluded from the efficacy analysis. The median monthly seizure frequency was 10.0 (IQR = 5.5–30.6) at baseline and 3.5 (IQR = 0–9.7) at LFV, with a corresponding median percentage reduction in baseline seizure frequency of 69.5% [IQR = 69.5 (20.8–98.2)]. At LFV, 29 out of 49 patients (59.2%) reported a ≥50% reduction in baseline seizure frequency with a mean CNB dose of 201.8 mg/day (25–400 ± 96.9), 42% of patients (n = 20) achieved ≥75% reduction, and 10 patients (20.2%) achieved seizure-freedom (Figure 1A). Six (13.3%) patients remained seizure-free and 4 (8.9%) experienced no more than one single seizure during at least 6 months. Responder rates over the 0–3 months, 3–6 months, 6–9 months, and 9–12 months intervals were 53.1%, 61.2%, 67.3%, and 65.3% respectively (Figure 1B). A low-CNB dosage (<200 mg per day) was used 17/49 patients (34.7%). The proportion of patients who experienced a 100%, ≥75%, or ≥50% reduction in seizure frequency at the last visit was not statistically significantly different in those receiving low-dose CNB (19.4%, 41.7%, and 58.3%, respectively) vs. those receiving high-dose CNB (20.3%, 45.5%, and 62.3%, respectively).

At 6 months, treatment retention for CNB was 100.0% (49/49), at 9 months, 81.6% (40/49, 6 patients with insufficient follow-up, 2 patients for lack of efficacy, 1 patient for AEs), and at 12 months, 95.2% (20/21 patients, 19 pts with insufficient follow-up, 1 patient for AEs). Binomial logistic regression failed to reveal any association among the efficacy outcome (seizure responder) and other parameters. Demographic and clinical characteristics were analyzed in responders and non-responders. We did not find significant differences in these two groups (see Supplementary Table).

### Concomitant ASMs and pharmacokinetics evaluations and drug-drug interactions during cenobamate use in clinical practice

We analyzed the potential effect of concomitant ASMs on the efficacy outcomes. The coadministration of sodium-channel blockers (SCBs) resulted in higher percentage of responders (64.1%) if compared with patients not taking SCBs (40.0%) (*p* = 0.003). Considering clobazam (CLB), responders were 66.7% among patients taking CLB compared with 55.9% among patients not taking CLB as concomitant medication (*p* = 0.05) (Figure 1C). In thirty-two (59.2%) cases out of 54 patients we were able to reduce or withdraw one or more concomitant ASMs (Table 2; Supplementary Figure S1).

**TABLE 2:** Concomitant ASMs modifications (*N* = 38).

Concomitant ASMs modifications	N (%)
Concomitant ASMs (type), n. of patients (%)	
CBZ	24 (44.4)
CLB	22 (40.7)
LCS	17 (31.5)
VPA	12 (22.2)
LTG	11 (20.3)
PB	11 (20.3)
PER	11 (20.3)
ZNS	6 (11.1)
BRV	6 (11.1)
LEV	5 (9.2)
OXC	4 (7.4)
RUF	3 (5.5)
TPM	3 (5.5)
GVG	3 (5.5)
CBD	3 (5.5)
PHT	1 (1.8)
ESL	1 (1.8)
CZP	1 (1.8)
NTZ	1 (1.8)
Patients who modified other ASMs with AEs	32/54 (59.2)
Patients who reduced 1 ASM	18/54 (33.3)
Patients who stopped 1 ASM	13/54 (24.0)
Patients who reduced/stopped 2 ASMs	1/54 (1.8)
Patients who reduced/stopped 3 ASMs	8/54 (14.8)
Patients who modified ASMs without AEs	7/54 (12.9)
Most frequent modified ASMs	
CBZ	7/54 (12.9)
LCS	5/54 (9.2)
CLB	8/54 (14.8)
LTG	3/54 (5.5)

N, number; ASMs, antiseizure medications; CNB, cenobamate; AEs, adverse events; CBZ, carbamazepine; CLB, clobazam; LCS, lacosamide; VPA, valproic acid; LTG, lamotrigine; PB, phenobarbital; PER, perampanel; ZNS, zonisamide; BRV, brivaracetam; LEV, levetiracetam; OXC, oxcarbazepine; RUF, rufinamide; TPM, topiramate; GVG, vigabatrin; CBD, cannabidiol; PHT, phenytoin; ESL, eslicarbazepine; CZP, clonazepam; NTZ, nitrazepam.

**FIGURE 2:**
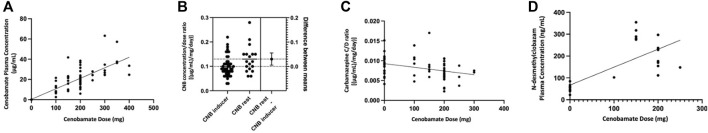
**(A)**. Significant (*p* < 0.0001) linear correlation (Pearson test) between the daily dose and the plasma concentration of Cenobamate in the dose range 100–400 mg. **(B)**. Difference in mean concentration/dose ratio of Cenobamate with and without concomitant use of at least one inducer antiseizure medication. **(C)**. Correlation between Cenobamate dose and carbamazepine concentration/dose ratio. **(D)**. Correlation between Cenobamate dose and N-desmethylclobazam plasma concentration.

Regarding CNB quantification in plasma, 65 measures were performed in 31 patients at different doses in the range of 100–400 mg/day (details are reported in Table 3). In this group of patients, we analysed the correlation (Pearson test) between CNB dose and plasma concentration (Figure 2A), and we found a significant linear correlation (*r* = 0.86, *p* < 0.0001). We further analysed the effect of concomitant administration of inducer ASMs on CNB plasma concentration. We found a significant difference in mean plasma concentration/dose administered ratio (C/D ratio) of CNB between patients assuming at least one inducer and patients without an inducer as concomitant medication (0.10 ± 0.04 [(μg/mL)/(mg/day)]; *n* = 47 vs*.* 0.13 ± 0.05 [(μg/mL)/(mg/day)]; *n* = 18, *p* = 0.04) (Figure 2B). As concerns the impact of CNB on the plasma concentrations of other ASMs, we have analysed CBZ plasma levels over time. Specifically, in 19 patients, a significant (dose-dependent) effect of CNB has been observed. Increasing doses of CNB were inversely correlated (*r* = -0.31, *p* = 0.02) to the C/D ratio of CBZ (Figure 2C). Similarly, CNB dose was positively correlated (*r* = 0.74, *p* < 0.0001) with an increased plasma concentration of the active CLB metabolite N-desmethylclobazam (N-CLB) (Figure 2D). CNB dose of 200 mg/day resulted in a 2-fold increase of N-CLB plasma levels (Supplementary Figure S2), notably, the increase in N-CLB should be further explored since our data suggest a potential non-linear correlation at increasing doses. Correlations among CNB dose and plasma concentrations of other concomitant ASMs were not significant. Notably, C/D ratios of levetiracetam (2 patients), valproate (6 patients) and zonisamide (4 patients) were partially reduced with CNB increasing doses. Among other ASMs, drug level of lacosamide (10 patients), phenobarbital (6 patients), lamotrigine (7 patients), and perampanel (6 patients) did not change.

**TABLE 3:** Cenobamate plasma concentration across different doses and single dose group concentration/dose (C/D) ratio.

Dose (mg)	Number of Measures	Plasma concentration μg/mL (median; range)	C/D [(μg/mL)/mg/day)] (median; range)
100	10	8.28 (2.55–21.56)	0.08 (0.03–0.22)
150	13	21.38 (8.49–41.78)	0.14 (0.06–0.28)
200	28	18.61 (5.96–38.49)	0.09 (0.03–0.19)
250	3	24.38 (19.18–27.87)	0.10 (0.08–0.11)
300	6	27.78 (24.45–63.21)	0.095(0.08–0.21)
350	3	37.61 (36.33–57.21)	0.11 (0.10–0.16)
400	2	29.09 (24.64-33-54)	0.07 (0.06–0.08)

### Neuropsychological evaluation

All patients had a general cognitive evaluation while 32 patients underwent the administration of a battery of neuropsychological tests at baseline and after 6 months since the start of treatment with CNB (Table 4). Administration of Raven’s Progressive Matrices 38 IQ showed a statistically significant improvement in total score (30.6 at baseline versus 41.56 at six-month evaluation, *p* = .05). General Anxiety Disorder-7 test shows a significant statistically improvement of final score (6.82 at baseline versus 4.53 at six-month evaluation, *p* = .03). A significant statistically improvement was observed also in TMT-A (68.31 at baseline versus 41.65 at six-month evaluation, *p* = .01) and ABAS II-DAP (91.59 at baseline versus 103.31 at six-month evaluation, *p* = .04). We did not find difference in mean GAD-7 final score responders versus non-responders (*p* = .08), moreover CNB dose and plasma levels did not correlate with GAD-7 final score (*r* = 0.41, *p* = 0.07 and *r* = 0.47, *p* = 0.09, respectively).

**TABLE 4:** Cognitive condition and neuropsychological evaluation.

	N (%)		
Cognitive condition (*N* = 54)			
No cognitive impairment	24 (44.4)		
Cognitive impairment	30 (55.5)		
Mild	11/30 (36.6)		
Moderate	12/30 (40.0)		
Severe	7/30 (12.9)		
Education (N = 54)			
No education	7 (27.8)		
Elementary school			
Middle school	3 (5.5)		
High school	26 (48.1)		
Graduation	10 (18.5)		
Neuropsychological tests (*N*= 32)	Baseline evaluation	Six months evaluation	*p*-value
Raven’s Progressive Matrices 38 IQ	30.57	41.56	**0.05**
Trail-making test A	68.31 s	41.65 s	**0.01**
Trail-making test B	142.67 s	146.94 s	0.90
Patient Health Questionnaire-9	6.27	7.53	0.37
General Anxiety Disorder-7	6.82	4.53	**0.03**
ABAS II—GAC	92.63	99.5	0.36
ABAS II—DAC	93.19	89.38	0.45
ABAS II—DAS	90.68	96.88	0.42
ABAS II—DAP	91.59	103.31	**0.04**
PedsQL Total	74.27	71.35	0.51
Health	79.33	76.94	0.6
Emotion	69.11	66.76	0.71
Socialization	76.61	75.59	0.89
School	67.78	64.41	0.56
Qolie 31 Total	49.88	49.88	0.99
Seizure Worry	49.04	46.35	0.36
Quality of life	48.42	51.29	0.34
Wellbeing	50.92	50.35	0.80
Energy	53.15	50.24	0.34
Cognition	51.35	51.06	0.92
Medical effects	51.58	49.71	0.50
Social functions	47.04	45.12	0.44

ABAS II, Adaptive Behavior Assessment System—Second Edition; GAC, general adaptive composite; DAC, conceptual skills domain; DAS, social skills domain; DAP, practical skills domain; PedsQL, pediatric quality of life inventory; Qolie 31, Quality of Life Inventory in Epilepsy. Bold values denote statistical significance at the *p* < 0.05 level.

### Safety

Data on safety were available for all patients (see Table 5). The most common AEs were somnolence (*n* = 17, 53.1%), dizziness (*n* = 9, 28.1%) and diplopia (*n* = 4, 12.5%). Dizziness and diplopia were more frequent in patients taking SCBs (36% and 16% versus 0% and 0% in patients without SCBs), while somnolence was more frequently reported by patients taking CLB (72.7%) compared with patients without CLB (42.8%) (Table 5). Somnolence was a temporary AE that most frequently resolved spontaneously, while the dose adjustment of concomitant ASMs or CNB was required in the other cases. The mean dose of CNB at the time of the occurrence of the AEs was 118.9 mg/day (12.5-250 ± 60.4). No patients presented SAEs or required hospitalization. AEs were observed in 63% of patients treated with low-dose CNB; 6.8% of patients discontinued treatment due to AEs.

**TABLE 5:** Adverse events reported (*N* = 54).

	N (%) Total*	Pts with SCB (44/54) N (%)	Pts without SCB (10/54) N (%)	Pts with CLB (22/54) N (%)	Pts without CLB (32/54) N (%)
Patients with AEs	32 (100)	25 (100)	7 (100)	11 (100)	21 (100)
Somnolence	17/32 (53.1)	12/25 (48)	5/7 (71.4)	8/11 (72.7)	9/21 (42.8)
Dizziness	9/32 (28.1)	9/25 (36)	0/7 (0)	4/11 (36.4)	5/21 (23.8)
Diplopia	4/32 (12.5)	4/25 (16)	0/7 (0)	1/11 (9.1)	3/21 (14.3)
Ataxia	3/32 (9.4)	2/25 (8)	1/7 (14.3)	0/11 (0)	3/21 (14.3)
Headache	2/32 (6.5)	1/25 (4)	1/7 (14.3)	1/11 (9.1)	1/21 (4.8)
Vomiting	1/32 (3.1)	1/25 (4)	0/7 (0)	0/11 (0)	1/21 (4.8)
Urticaria	1/32 (3.1)	1/25 (4)	0/7 (0)	0/11 (0)	1/21 (4.8)
Diarrhea	1/32 (3.1)	0/25 (0)	1/7 (14.3)	0/11 (0)	1/21 (4.8)
Outcome					
Resolved	21/32 (65.6)	10/25 (40)	3/7 (42.8)	4/11 (36.4)	11/21 (52.4)
Not resolved/ongoing	8/32 (25)	5/25 (20)	3/7 (42.8)	3/11 (27.3)	5/21 (23.8)
Resolved with ASMs modification	3/32 (9.4)	10/25 (40)	1/7 (14.3)	4/11 (36.4)	5/21 (23.8)
Severity					
Mild	16/32 (50)	14/25 (56)	2/7 (28.6)	4/11 (36.4)	12/21 (57.1)
Moderate	9/32 (28.1)	5/25 (20)	4/7 (57.1)	4/11 (36.4)	5/21 (23.8)
Severe	7/32 (21.9)	6/25 (24)	1/7 (14.3)	3/11 (27.3)	4/21 (19.0)
SAEs	0 (0)	0 (0)	0 (0)	0 (0)	0 (0)
Hospitalization	0 (0)	0 (0)	0 (0)	0 (0)	0 (0)
CNB dose at time AEs (mg/kg/day)	118.9 (12.5-250 ± 60.4)	120.5 (12.5-250 ± 64.7)	100.0 (50-150 ± 40.8)	131.8 (50-200 ± 51.3)	107.7 (12.5-250 ± 65.1)
EKG				ANOVA *p* = 0.47	
QTc baseline (mean, msec)		408.3			
QTc at 3 months (mean, msec)		401.4			
QTc at 6 months (mean, msec)		409.5			
QTC at 9 months (mean, msec)		405.5			

N, number; Pts, patients; SCB, sodium channel blockers; CLB, clobazam; AEs, adverse events; ASMs, antiseizure medications; SAEs, serious adverse events; CNB, cenobamate; EKG, electrocardiogram; QTc, Corrected QT, Interval (QTc) with Fridericia correction formula.

*Patients can have more AE.

Considering electrocardiogram monitoring, none of the patients experienced significant alterations of the QTc interval (ANOVA, *p* = .47). No significant blood tests alterations were registered.

## Discussion

In this retrospective, real-world study of CNB treatment in patients with drug resistant focal epilepsies, add-on CNB provided a clinically significant reduction in seizure frequency in most of the patients. The results we found in the current study are consistent with those observed in previously published randomized controlled trials (RCTs) (Chung et al., 2020; Krauss et al., 2020) and real-life experiences (Elliott et al., 2022; Makridis et al., 2022; Varughese et al., 2022; Beltrán-Corbellini et al., 2023; Friedo et al., 2023; Peña-Ceballos et al., 2023; Villanueva et al., 2023).

Of note, this study allowed to obtain more information about the use of CNB in the context of the real-world practice, adopting a more flexible drug dosing regimen and providing data over a longer follow-up period if compared to earlier RCTs.

The median percentage reduction of seizure frequency of 69.5% and the responder rate of 59.2% were slightly higher compared to those reported in both pivotal studies: in C013 and in C017 trials, the reductions in median seizure frequency were 55.6% and 56% and the responder rates were 50.4% and 56% at CNB dose of 200 mg/day, respectively (Chung et al., 2020; Krauss et al., 2020; Lattanzi et al., 2020). The proportion of seizure-free patients in the present study was 20.4% at a mean CNB dose of 200 mg/day. This figure is lower if compared with the rate found in the C013 study (28% at maintenance dose of CNB 200 mg/day) (Chung et al., 2020), and higher than the rate reported in the C017 study (11% at maintenance dose of CNB 200 mg/day) (Krauss et al., 2020). Considering the different study designs, results in our cohort of patients seem to be slightly better if compared with the previous RCTs. This can be explained by the possibility of adjusting and personalizing the dose of CNB and concomitant ASMs, which may have given higher responder rates.

The results of this study were similar to results from other real-world studies of CNB, which also included patients with highly refractory epilepsy.

In a recent large series of patients with highly drug-resistant epilepsy, including 170 patients, the rate of seizure freedom was 13.3%; ≥90%, ≥75%, and ≥50% responder rates were 27.9%, 45.5%, and 63%, respectively. There was a significant reduction in the number of seizures per month (mean: 44.6%; median: 66.7%) between baseline and the last visit (*p* < 0.001) (Villanueva et al., 2023). Compared with this study (Villanueva et al., 2023), we observed a higher percentage of seizure-free patients (13.3% vs. 20.4%). This could be due to differences in the cohort composition, since our patients were slightly younger, and a lower number of previous treatments, suggesting that CNB could be associated with a better response when used earlier. Of note, we also found that 8.9% of patients were nearly to seizure freedom (patients with no more than one seizure during six consecutive months of the study period).

From a series of 45 adolescent and adult patients who had received a mean of 12 prior ASMs, Elliot et al. reported seizure freedom in 16% of patients and a ≥50% response in 60% (Elliot et al., 2022). A pediatric real-world study found a seizure freedom rate of 31% and a >50% response rate of 37.5% (Makridis et al., 2022). In another pediatric real-world study, 19% of patients achieved seizure freedom, 52% had a ≥75% response rate, and 63% had a ≥50% response rate (Varughese et al., 2022). Additionally, our results were in line with previous findings from an EAP in Ireland, which 57 patients with ultraresistant epilepsy and showed seizure reduction in 75%–99% of the cohort in 42.1% of patients (Peña-Ceballos et al., 2022).

More recently, in the Beltran-Corbellini et al. study, in 51 patients with highly refractory focal epilepsy, retention rate at the last follow-up was 80.4% and the 50% responder rate in focal seizures was 56.5% and in focal to bilateral tonic-clonic seizures was 63.6% (Beltran-Corbellini et al., 2023).

Finally, a drug load reduction was possible in 12 patients with developmental and epileptic encephalopathies, thanks to CNB effectiveness, maintaining seizures reduction (Friedo et al., 2023).

Considering the responder rates, we found some differences in patients with and without concomitant SCBs: the concomitant use of SCBs increased the percentage of seizure free patients from 10% to 23% and the percentage of responders from 40% to 64%. Concomitant use of SCBs and CNB, should be carefully evaluated for tolerability issues. Although a similar increase of responders was seen in patients with and without concomitant CLB (67% versus 56%), the seizure freedom rate was higher among patients not taking concomitant CLB (26.5% versus 6.7%); we do not have a clear-cut explanation for this latter finding, which should be further investigated in future studies.

The longitudinal evaluation of efficacy revealed an increase in the responder rates from the first to the third trimester, going from 53% to 67.3% (≥50% responders) and from 8.2% to 18.4% (seizure freedom). An improvement of the drug efficacy is seen over time, possibly related also to the increase in drug dosage, and clinicians should wait before considering CNB withdraw if the tolerability is kept, even if we have to acknowledge that—as it always happens in long term observation—the overall number of patients during time decrease making the results more attractive.

Reducing the load of concomitant ASMs is highly warranted in patients with drug resistant epilepsy: in the study cohort, most patients were taking a median of 3 ASMs and still experiencing a median of 11.7 seizures per month. In this regard, the 60% of the participants were able to reduce or withdraw one or more concomitant ASMs during the follow-up.

The incidence of AEs was higher if compared with literature data, even though a clear association with a specific CNB dose was not identified. Most AEs occurred at a relatively low dose of CNB (100 mg/day) and without the dose-related association registered in previous clinical trials (Chung et al., 2020; Krauss et al., 2020). A possible explanation of the poorer tolerability may be the use of multiple concomitant ASMs in our cohort of patients. Central nervous system-related side effects were the most reported as already found with the use of other currently available ASMs (Zaccara et al., 2013). Ataxia and diplopia were more frequent when a concomitant SCB was taken, likely as the result of the pharmacodynamic interaction that may occur among agents acting as SCBs.

In many cases, a reduction in the number and/or dose of concomitant ASMs was required to resolve AEs. SCBs and CLB were the most frequently modified concomitant drug regimens. A proactive lowering of ASMs with known pharmacodynamic (carbamazepine and lacosamide) and pharmacokinetic interactions (phenobarbital, phenytoin, CLB) with CNB may be indicated to prevent AEs and allow an optimal CNB dosing (Smith et al., 2022).

A linear correlation was shown between the daily dose and the plasma concentration of CNB in the dose range 100–400 mg/day. Dose-proportional increases in both maximum concentration and plasma exposure are confirmed by single and multiple ascending dose studies (Vernillet et al., 2020), but non-linear pharmacokinetics is expected at higher doses (more than 300 mg). In our cohort, the number of patients treated with more than 300 mg of CNB was too small to assess any disproportionality in dose responses.

Plasma levels measurements confirm that CNB acts both as “victim” and as “perpetrator” of drug-drug interactions. Indeed, in patients treated with at least one inducer, the mean C/D ratio of CNB was significantly lower if compared with patients not taking an inducer. On the other hand, the reduction of CBZ plasma concentrations induced by CNB concomitant administration (Roberti et al., 2021) has been confirmed in our cohort. Also, the pharmacokinetic interactions involving CLB, and its metabolite have been confirmed, considering that we observed a 2-fold increase in N-CLB levels at CNB 200 mg/day. Such increase of N-CLB was associated with somnolence in our patients. Notably, a variable (between 145% and 1852%) increase in N-CLB serum concentration has been reported in a recent study (Elakkary et al., 2023). The authors suggest a potential role of the pharmacogenetics of the hepatic enzyme CYP2C19 to explain this high rate of variability and report fatigue as a direct consequence of N-CLB increase (Elakkary et al., 2023). In the same study, a synergistic interaction between CLB and CNB has been hypothesized, because both ASMs potentiate GABA transmission acting at different sites of the GABA_A_ receptor (Elakkary et al., 2023).

Most notably, there was a certain degree of variability within the same dose among patients, therefore, TDM is likely recommended to better adjust therapy. This is also in agreement with our observation of the reduction of CNB concentration when inducer ASMs are concomitantly used, and the bidirectional influence observed.

The lack of a significant correlation among CNB doses and plasma concentrations of other concomitant ASMs may be due to the overall limited number of observations.

General Anxiety Disorder-7 test showed a significant improvement in symptom severity of anxiety score in our cohort. This result could be related to the reduction of seizures burden or to the CNB pharmacodynamic mechanism as positive allosteric modulator of the GABA_A_ ion channel (Löscher et al., 2021). We failed to find any correlations between reduction of anxiety, the status of responder, and CNB blood concentrations. More studies should be performed to document and confirm if the reduction of anxiety is related to seizure’s reduction or to the GABA_A_ modulation. We observed an improvement in Raven’s Progressive Matrices 38 IQ, TMT-A and ABAS-II probably due to the reduction of the concomitant drug load.

### Study limitations

The limitations of this study are: the small sample size which does not allow internal statistical comparison; a potential selection bias considering the nature of patients selection, although, being an EAP, the selected patients represent a subgroup of patients with a high number of previous ASMs failures; the relatively short follow-up which does not allow the evaluation of long-term efficacy above all considering seizure freedom; the open-label design, the retrospective nature, and the lack of a control group.

## Conclusion

If confirmed by future studies, the efficacy shown by CNB in reducing seizures in patients with FOS may support the indication to use CNB sooner in the treatment algorithm of focal epilepsy. We reported that lower doses of CNB may be as effective as those used in previous RCTs. Moreover, the use of add-on CNB allowed to reduce the overall load of concomitant ASMs.

Therapeutic drug monitoring could be a useful tool to improve CNB dosing and prevent possible AEs. CNB appears to reduce symptom severity of anxiety.

CNB was safe and provided sustained, clinically meaningful seizure reduction in this real-world study. More studies may better establish the long-term safety and efficacy of CNB and identify possible predictors of seizure control.

## Data Availability

The raw data supporting the conclusion of this article will be made available by the authors, without undue reservation.
