# Supplementary material for: Cenobamate as add-on therapy for drug resistant epilepsies: effectiveness, drug to drug interactions and neuropsychological impact. What have we learned from real word evidence?
Source: Front Pharmacol. 2023 Dec 21;14:1239152. doi: 10.3389/fphar.2023.1239152 (PMC10768012; doi:10.3389/fphar.2023.1239152)
Supplement: Supplementary file 1 [file Table1.docx]

**Supplementary Table 1. Demographics and clinical features in responders and non-responders**

|  | **Responders**  **N (%) or Mean (ranges ± SD) or Median (Q1, Q3)** | **Non-responders**  **N (%) or Mean (ranges ± SD) or Median (Q1, Q3)** |
| --- | --- | --- |
| Patients | 29 | 20 |
| Sex  Male  Female | 12 (41.4)  17 (58.6) | 10 (50.0)  10 (50.0) |
| Age at epilepsy onset (years) | 6.3 (2.0, 9.0) | 5.8 (2.5, 8.3) |
| Age at enrolment (years) | 25.4 (20.2, 31.7) | 26.9 (22.9, 31.4) |
| Disease duration (years) | 23.5 (15.7, 28.1) | 20.8 (15.7, 24.9) |
| Etiology  Unknown  Genetic  Genetic/Structural (TSC)  Structural  Infective  Autoimmune | 10 (34.4)  1 (11.1)  3 (10.3)  13 (44.8)  2 (6.9)  0 (0.0) | 5 (25.0)  3 (15.0)  0 (0.0)  11 (55.0)  0 (0.0)  1 (5.0) |
| Previous SE | 4 (13.9) | 3 (15.0) |
| Follow-up (months) | 10.9 (6.0-15.0 ± 1.9) | 10.1 (1.0-14.0 ± 3.1) |
| Dose of CNB at last follow-up (mg/day) | 112.5 (50-400 ± 98.7) | 216.25 (25-400 ± 85.9) |
| Weight (kg) | 69.3 (30.0-121.0 ± 18.5) | 64.4 (45.0-85.0 ± 11.6) |
| Previous idiosyncratic reactions | 2 (6.9) | 2 (10.0) |
| Previous ASMs | 8 (7, 11.2) | 10 (8, 12) |
| Concomitant ASMs (number) | 1.5 (2, 3) | 3 (2, 3.2) |
| Other treatments  VNS  Neurosurgery  KD | 7 (24.1)  11 (37.9)  0 (0.0) | 5 (25.0)  9 (45.0)  0 (0.0) |
| Seizure type (n, %)  Focal  Focal to bilateral | 29 (100.0)  9 (31.0) | 20 (100)  7 (35.0) |
| Other types of seizures  Tonic  Atonic  Atypical absences  Spasms | 2 (6.9)  2 (6.9)  0 (0.0)  0 (0.0) | 3 (15.0)  1 (5.0)  2 (10.0)  1 (5.0) |
| Baseline seizure frequency  Mean  Median | 22.7 (2-137 ± 32.7)  8.5 (4.0, 26.9) | 22.6 (3.0-65.0 ± 17.5)  16 (9, 32.2) |
| Titration period (days) at 100 mg/day | 57.2 (25-140 ± 17.2) | 59.1 (20-162 ± 15.8) |
| Titration period (days) at 200 mg/day | 108.2 (46.2-250.1 ± 38.32 | 105.2 (77.1-282.3 ± 43.3) |
| Titration period (days) at CNB maximum dose | 151.8 (15.1-450 ± 105.1) | 172.8 (13.3-422.1 ± 108.2) |
| CNB withdraws (n, %)  Adverse events  Inefficacy  Increased seizure frequency | 1 (3.4)  0 (0.0)  1 (3.4)  0 (0.0) | 3 (15.0)  2 (10.0)  1 (5.0)  0 (0.0) |

*N, number; SD, standard deviation; Q1, first quartile; Q3, third quartile;* TSC, tuberous sclerosis complex; SE, status epilepticus; CNB, cenobamate; *ASMs, antiseizure medications; VNS, vagal nerve stimulation; KD, ketogenic diet.*

**Supplementary Table 2. Cenobamate plasma concentration and dose for every single patient included in the pharmacokinetinc analysis of cenobamatedose/plasma concentration correlation**

| **Patient ID** | **Cenobamate Daily Dose (mg)** | **Plasma Concentration (μg/mL)** |
| --- | --- | --- |
| 1AA | 200 | 12.65 |
| 1AA | 300 | 28.87 |
| 1AA | 300 | 34.62 |
| 2AB | 200 | 15.29 |
| 2AB | 300 | 24.57 |
| 2AB | 300 | 63.21 |
| 3AC | 200 | 17.04 |
| 4AD | 200 | 17.01 |
| 4AD | 200 | 21.72 |
| 4AD | 250 | 24.38 |
| 5AE | 200 | 19.58 |
| 5AE | 300 | 26.68 |
| 6AF | 100 | 8.22 |
| 6AF | 200 | 22.25 |
| 6AF | 200 | 22.73 |
| 7AG | 200 | 28.21 |
| 7AG | 200 | 38.49 |
| 7AG | 250 | 27.87 |
| 8AH | 150 | 22.39 |
| 9AI | 200 | 18.81 |
| 10AJ | 100 | 2.55 |
| 11AK | 150 | 8.49 |
| 11AK | 150 | 8.67 |
| 11AK | 150 | 8.67 |
| 12AL | 100 | 8.34 |
| 12AL | 100 | 6.32 |
| 12AL | 100 | 7.03 |
| 13AM | 200 | 11.76 |
| 13AM | 200 | 18.23 |
| 13AM | 200 | 18.41 |
| 14AN | 300 | 24.45 |
| 15AO | 200 | 31.52 |
| 15AO | 200 | 34.35 |
| 16AP | 150 | 18.73 |
| 16AP | 350 | 36.33 |
| 17AQ | 150 | 21.38 |
| 17AQ | 150 | 41.78 |
| 18AR | 100 | 13.50 |
| 18AR | 150 | 23.25 |
| 19AS | 200 | 15.08 |
| 19AS | 200 | 16.69 |
| 20AT | 200 | 16.09 |
| 20AT | 200 | 20.62 |
| 21AU | 150 | 10.06 |
| 21AU | 150 | 15.88 |
| 21AU | 200 | 5.96 |
| 22AV | 100 | 12.14 |
| 22AV | 150 | 22.42 |
| 23AW | 200 | 18.10 |
| 23AW | 350 | 57.21 |
| 23AW | 400 | 33.54 |
| 24AY | 200 | 20.04 |
| 24AY | 200 | 22.23 |
| 25AX | 200 | 10.33 |
| 26AZ | 200 | 23.98 |
| 26AZ | 250 | 19.18 |
| 27BA | 100 | 7.27 |
| 28BB | 200 | 11.82 |
| 28BB | 350 | 37.61 |
| 28BB | 400 | 24.64 |
| 29BC | 200 | 36.74 |
| 30BD | 100 | 21.56 |
| 31BE | 100 | 18.71 |
| 31BE | 150 | 22.58 |
| 31BE | 150 | 23.1 |

***Supplementary Figure 1.*** *Number of patients who discontinued, reduced or did not change concomitant antiseizure medications (ASMs) during CNB treatment.*

*
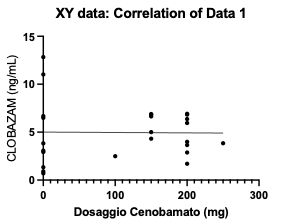
*

***Supplementary Figure 2.*** CNB dose of 200 mg/day resulted in a 2-fold increase of N-CLB plasma levels
