# Supplementary material for: Cenobamate as add-on therapy for drug resistant epilepsies: effectiveness, drug to drug interactions and neuropsychological impact. What have we learned from real word evidence?
Source: Front Pharmacol. 2023 Dec 21;14:1239152. doi: 10.3389/fphar.2023.1239152 (PMC10768012; doi:10.3389/fphar.2023.1239152)
Supplement: Supplementary file 2 [file DataSheet1.PDF]

Supplementary Methods

A stock solution of cenobamate was prepared at 500 µg/mL in Milli-q pure water. Liquid chromatography (LC) was performed by using the chromatographic column and mobile phases included in the ClinMass ® LC-MS/MS Antiepileptic Drugs Complete Kit, (Recipe ® Chemicals & Instruments, GmbH). Deuterated retigabine (retigabine-D4), contained into the internal standard mix included in the ClinMass ® kit, was used as internal standard (IS). The analytical run time for each injection was 5 min, including 1.3 min of re-equilibration. The injection volume was 1.0 µL. Chromatographic conditions were set up following instructions provided by ClinMass ® kit. Calibration curve for cenobamate was linear and ranged from 1.0 to 50.0 µg/mL as previously reported (Yang E et al., 2022; Vernillet L et al., 2020). The lower limit of quantification (LLOQ) was 1.0 µg/mL. Two quality controls (QCs) were prepared at low (L-QC) and high (H-QC) concentration of 3.0 and 30.0 µg/mL respectively. Cenobamate concentrations in plasma samples were determined as follows: plasma was recovered by centrifuging EDTA blood samples at 3,500xg for 5 minutes. Thereafter, 50 µL of each calibrator, QC and plasma sample were transferred into eppendorf tubes (Eppendorf s.r.l, 20159 Milan, Italy) and mixed with 100 µL of internal standard mix containing retigabine-D4 (provided by kit ClinMass ® kit). After mixing for 30 seconds and centrifuging at 13,000 rpm for 9 minutes at room temperature, 100 µL of supernatant from each tube were transferred to vials and injected into LC- MS/MS system. Mass spectrometric conditions were as follows: Gas Temperature 325°C, Gas Flow 9 l/min, sheath Gas temperature 400°C, Sheath Gas Flow 12 l/min, Capillary 2000 V, Nebulizer 40 psi. Samples were detected in multiple reaction monitor (MRM) mode. Mass transitions for cenobamate were: m/z 268.1→ 198.0 for quantifier and 268.06 →155.0 for qualifier; mass transition for retigabine-D4 was: m/z 308.2→ 113.0. The software used for controlling this system and analyzing results was MassHunter (Agilent Technologies). Drug-free plasma was obtained

from healthy volunteers recruited at the Blood Transfusion Center of the Children's Hospital Bambino Gesù after obtaining informed consent, and was used as a matrix for standard curve preparation and negative controls. Prior to use, cenobamate stock solutions and human blank plasma were stored at -80 and −20 °C, respectively. Method validation was carried out according to the current European Medicines Agency guidelines on validation of bioanalytical methods (European Medicines Agency. Guideline on Bioanalytical Method Validation. Available at: https://www.ema.europa.eu/en/documents/scientific-guideline/guideline- bioanalytical-method-validation_en.pdf. Accessed on October 4, 2022). Specifically, linearity, accuracy and precision, specificity and selectivity, carry-over and stability were evaluated. All other ASMs were quantified when clinically appropriate and requested according to standardized clinical laboratories methods.
